# Egg Production in a Coastal Seabird, the Glaucous-Winged Gull (*Larus glaucescens*), Declines during the Last Century

**DOI:** 10.1371/journal.pone.0022027

**Published:** 2011-07-18

**Authors:** Louise K. Blight

**Affiliations:** Centre for Applied Conservation Research, University of British Columbia, Vancouver, British Columbia, Canada; Institute of Marine Research, Norway

## Abstract

Seabirds integrate information about oceanic ecosystems across time and space, and are considered sensitive indicators of marine conditions. To assess whether hypothesized long-term foodweb changes such as forage fish declines may be reflected in a consumer's life history traits over time, I used meta-regression to evaluate multi-decadal changes in aspects of egg production in the glaucous-winged gull (*Larus glaucescens*), a common coastal bird. Study data were derived from literature searches of published papers and unpublished historical accounts, museum egg collections, and modern field studies, with inclusion criteria based on data quality and geographic area of the original study. Combined historical and modern data showed that gull egg size declined at an average of 0.04 cc y^−1^ from 1902 (108 y), equivalent to a decline of 5% of mean egg volume, while clutch size decreased over 48 y from a mean of 2.82 eggs per clutch in 1962 to 2.25 in 2009. There was a negative relationship between lay date and mean clutch size in a given year, with smaller clutches occurring in years where egg laying commenced later. Lay date itself advanced over time, with commencement of laying presently (2008–2010) 7 d later than in previous studies (1959–1986). This study demonstrates that glaucous-winged gull investment in egg production has declined significantly over the past ∼50–100 y, with such changes potentially contributing to recent population declines. Though gulls are generalist feeders that should readily be able to buffer themselves against food web changes, they are likely nutritionally constrained during the early breeding period, when egg production requirements are ideally met by consumption of high-quality prey such as forage fish. This study's results suggest a possible decline in the availability of such prey, and the incremental long-term impoverishment of a coastal marine ecosystem bordering one of North America's rapidly growing urban areas.

## Introduction

Life history theory predicts that long-lived organisms such as seabirds will maximise fitness by reducing reproductive output during periods of environmental stress, trading off between current and future reproduction. One potential way for birds to reduce reproductive investment when foraging conditions are poor early in the breeding season is by decreasing the size or number of eggs produced. Female protein and energy requirements during egg production are substantially higher than those during the non-laying period, making egg production costly [1]–[3] (but see [4]). Indeed, for many avian species there is strong evidence that under poor food conditions, egg size, number or both are reduced, though lay date responds to food supply more consistently than do egg or clutch size [5]–[7]. Trade-offs reduce reproductive performance in a given year; therefore, repeated poor years, for example due to environmental factors including climatic variation and/or competition with humans for prey [8], can mean that adult survival is traded off against a better future that never materialises, with population numbers ultimately affected. Thus, ongoing poor conditions will also have long-term population consequences, and understanding the mechanisms driving such changes can have important conservation implications [9].

The world's oceans are now strongly affected by human activities, with most marine food webs simplified and impoverished by drivers such as pollution, climate change, and overfishing [10]. Like many other coastal areas over the last century or more, the inshore waters of southern British Columbia (BC) and northern Washington (WA; hereafter, the Salish Sea) have seen removal of upper trophic predators such as whales and sequential overfishing of forage fishes such as Pacific herring (*Clupea pallasii*) [11]. This, in combination with other factors such as climate change and pollution, means that this area is now among those globally estimated to be suffering very high levels of human impacts [10]; thus, ecosystem productivity and function in the region is potentially very different than it was prior to the start of industrial activity [11]–[13]. Marine systems worldwide have responded in varying ways to removal of predators and prey [14], [15], and as common meso-predators, marine birds are considered to be sensitive indicators of such changes in oceanic food webs, particularly given the long-term nature of some colonial seabird studies (e.g., [16]). The glaucous-winged gull (*Larus glaucescens*) is a conspicuous marine bird that breeds at accessible coastal nesting colonies in the northern Pacific, and as such it represents a strong potential source of indicator data: ecologists and naturalists have been researching its reproductive biology, conducting colony counts, and collecting its eggs for museums for over 100 years.

During the nesting season glaucous-winged gull diet in the study area consists of small forage fishes such as herring and sandlance (*Ammodytes hexapterus*), trash, and invertebrates, with diet currently (2008 – present) appearing to consist primarily of marine foods [17], [18]. Though trash is frequently available, it is not clear whether it is beneficial to gulls. In some parts of the world gull populations have declined in apparent response to the covering of landfills and loss of anthropogenic foods [19], and trash has also been implicated in glaucous-winged gull population trends in the Salish Sea [17]. However, glaucous-winged gulls eating only herring were able to raise larger broods than were those whose diet included trash [20], and for congeneric Western gulls (*L. occidentalis*) the most successful breeders avoided eating refuse and instead fed themselves and their young mainly on fish prey [21]. Reduced productivity and poorer body condition was also documented in breeding female herring gulls (*L. argentatus*) that subsisted primarily on a trash-based diet relative to those subsisting primarily on fish foods [22]. The availability of Pacific herring, currently the principle forage fish in the Salish Sea, has likely declined in recent years, with factors such as pollution, climate change and historical overfishing believed responsible; herring were heavily exploited as early as 1910 and a stock collapse occurred in the 1960s [11]. Although some regional herring populations increased between about 1970 and 2002, others have decreased by up to two orders of magnitude over this period [13], [23]–[25]. Herring size-at-age has also declined since the 1970s at various eastern Pacific sites including the Salish Sea [13], indicating a potential decrease in food value of individual forage fish (cf. [26]). In addition, the spatial and temporal extents of spawning events in at least some parts of the study area have been decreasing, with a contraction of locations since the late 1980s, and a loss of early (January – early February since about 1970) and late (April–May since the early 1980s) spawners (Fig. 2 in [13]). In the Salish Sea, glaucous-winged gulls begin to arrive at their colonies in February and commence egg laying in mid- to late May.

The purpose of this study was to assess whether hypothesised long-term food web changes in this relatively under-studied coastal ecosystem might affect a consumer's life history traits over time. Because large-bodied single brooded birds obtain the resources necessary for egg production in advance of the breeding season as well as during it (i.e., they are primarily “capital” breeders; [6], [27]), and as the nutritional and energetic costs of egg production seem to be relatively high in larids [1], [28], [29], I predicted that glaucous-winged gulls would be sensitive to long-term decreases in food availability prior to the breeding season as well as during egg formation, and that they would respond to this by decreasing egg or clutch size over time. To test this prediction I used a meta-analytical approach and multiple data sources, including published records and museum egg collections, to examine long-term trends in egg (108 years) and clutch (48 years) sizes. Because clutch size progressively decreases with lay date in most single-brooded species [6], I also tested whether clutch size was correlated with timing of breeding in the study population. Researchers often record avian clutch size and lay date, and a number of studies have used longitudinal data to report long-term trends in these traits. Egg size has been studied less often, however, with few studies reporting long-term patterns in egg size variation [30]–[32]. Though avian eggs have been collected by naturalists and biologists for about 200 years, no studies have yet used museum collections to report on long-term trends in egg size (but see [33]). Lastly, because food availability is believed to influence lay date in bird species more consistently than it affects egg production [7], I also investigated changes in timing of breeding (over 52 y), predicting that if overall food availability had decreased in the Salish Sea this would result in delayed lay dates.

## Methods

### Study area

Field data for these analyses came from studies carried out between 1902 and 2010 at glaucous-winged gull colonies in the Salish Sea, i.e., the inshore coastal waters of the Strait of Georgia, BC, Canada, and adjacent waters, including northern Puget Sound, WA, USA and the adjacent eastern Strait of Juan de Fuca (range: 47.91°–50.02° N, 121.95°–125.24° W). Earlier banding studies, physical geography, and patterns of hybridization support the selection of this entire region, as does the colonies' shared history of 19^th^ century exploitation and subsequent recovery [34], [35] and their modern existence on the edge of some of the most rapidly-growing areas in Canada [36]. These boundaries ensured that I included all of the large colonies found in the region's inshore sea, but excluded the more westerly colonies that are strongly influenced by the open Pacific Ocean.

### Data sources and inclusion criteria

I compiled published data on glaucous-winged gull egg size, clutch size and first egg date obtained from a literature search using ISI Web of Science and keywords “glaucous-winged gull” and “Larus glaucescens”, the sources provided in the Birds of North America species account [37], and additional references cited in publications located via these searches. “Grey literature” (e.g., government reports) was included in these citations, and incorporated into the study accordingly. I applied no English-language or publication year restrictions. In the literature search, I included publications on glaucous-winged gulls that were not specifically about their reproduction because some authors (particularly in papers and reports prior to 1960, presumably as a result of older stylistic conventions) included appendices of miscellaneous biological data on the species. I supplemented published data with those I collected from 2008 to 2010 at Mandarte Island, BC (48.63°N, 123.28°W) and Arbutus Island, BC (48.70°N, 23.43°W), using methods comparable with those from earlier studies. For egg size, I also searched museum databases (ORNIS and institutions' own records) for egg sets collected from the study area, and obtained egg length and width measurements from five museum collections (specific museums listed in Acknowledgements section). To ensure that the published studies had taken place in the study area, I screened them by geographical region and then reviewed them against inclusion criteria related to research design and reporting of data (below).

In screening published studies of egg size, I only included those reporting measurements for entire clutches, i.e., those where every egg in a nest was measured. I excluded egg measurements for 2-egg clutches, reported separately in all studies, because eggs from these clutches are smaller on average than those from the modal 3-egg clutch [37] and the proportion of 2-egg clutches reported varied by study. I only included annual egg size means (from published studies and museum specimens) derived from more than a single clutch, and assumed that eggs collected by museums represented a random subsample of those available at a given colony because their volumes showed an approximately normal distribution, i.e., data were non-skewed. For clutch size, I required that studies had monitored their nests throughout a colony every 1–2 d for the duration of the laying period, i.e., I excluded studies reporting clutch sizes from opportunistic colony visits because clutch size is variable over the season. Two early studies provided no data but stated that “normal” clutch size was three (with 2-egg clutches “occasionally” found; [38], [39]); as I encountered only one actual measurement of clutch size prior to the 1980s, I retained these additional studies for comparative purposes and considered that their estimates represented a clutch size of 2.8, but did not include them in the analysis itself. Similarly, I required that published data on first egg date were collected using systematic colony monitoring protocols rather than opportunistic visits. All author-collected egg size, clutch size and lay date data (i.e., those I collected from 2008–2010; see above) were collected so as to be consistent with these literature-screening criteria.

No studies needed to be discarded due to a lack of essential meta-analytical data such as sample size. After screening of published studies and museum specimens, and addition of author-collected data, I ended up with five separate studies from which I derived seven annual means of egg size, as well as measurements from 329 eggs held in museum collections, representing an additional 14 annual means of egg size (“egg-years”; *n* = 21); each egg-year was treated as a sample unit (Table 1). These egg size data spanned more than a century (1902–2010) and represented at least 14 glaucous-winged gull colonies in the study area. I did not consider study area localities that had been recorded by museum collectors as “unnamed” to be additional colonies. I retained six studies from four Salish Sea colonies reporting nine annual mean clutch sizes, and 18 estimates of first egg date from six colonies (Table 1). As with egg size, each clutch-year was a sample unit. For a measure of timing of breeding, I chose first egg date rather than median lay date because my nest search effort was consistent through to the late laying season, but did not continue for long enough to record the latest nests; other included studies appeared to have followed a similar protocol. While first egg date is probably more subject to stochastic variation or sampling error than is median lay date, it is nonetheless considered a reliable indicator of timing of breeding [40]. All annual means were independent (i.e., they were not collected as repeated measures series at study sites), and as study sites were all located in or around the same inland body of water (the Salish Sea) I assumed no effect of site on vital rates, based on published inter-site comparisons of these parameters [41]–[44]. Standard meta-analyses address the possibility of publication bias (publication of studies showing an effect vs. non-publication of those showing no effect) but as my study simply assessed mean measures of egg production, consideration of such bias was unnecessary.

**Table 1:** Summary of studies used in standard and meta-analyses.

Num.	Data source	Nesting colony	Location	Year(s) data collected	Response variable	*N* (effect size estimates)
1	Museum collections^1^	Various^2^	Throughout study area^3^	1902–1946	Egg size	14
2	Schultz 1951	San Juan Islands	Puget Sound/Strait of Juan de Fuca, WA^4^	1948	Egg size	1
3	James-Veitch & Booth 1954	Williamson Rock	Puget Sound/Strait of Juan de Fuca, WA	1951	Egg size	1
4	Drent et al. 1962	Mandarte Island	Haro Strait, BC^5^	1959, 1960	Lay date	2
5	Vermeer 1963	Mandarte Island	Haro Strait, BC	1961, 1962	Lay date	2
6	Vermeer 1963	Mandarte Island	Haro Strait, BC	1962	Clutch size	1
7	Hunt & Hunt 1976	Mandarte Island	Haro Strait, BC	1971, 1973	Lay date	2
8	Verbeek 1986	Mandarte Island	Haro Strait, BC	1976, 1977, 1979, 1980	Lay date	4
9	Verbeek 1986	Mandarte Island	Haro Strait, BC	1979, 1980	Clutch size	2
10	Verbeek 1986	Mandarte Island	Haro Strait, BC	1980	Egg size	1
11	Reid 1987	Protection Island	Strait of Juan de Fuca, WA	1984	Lay date	1
12	Vermeer 1988	Vancouver Harbour	Strait of Georgia, BC	1986	Lay date	2
13	Vermeer 1988	Vancouver Harbour	Strait of Georgia, BC	1986	Clutch size	2
14	Vermeer 1988	Vancouver Harbour	Strait of Georgia, BC	1986	Egg size	2
15	Hooper 1988	Victoria Harbour	Strait of Juan de Fuca, BC	1986	Lay date	1
16	Hooper 1988	Victoria Harbour	Strait of Juan de Fuca, BC	1986	Lay date	1
17	LK Blight, unpubl data	Mandarte Island	Haro Strait, BC	2008–2010	Lay date	3
18	LK Blight, unpubl data	Mandarte Island	Haro Strait, BC	2008, 2009	Clutch size	2
19	LK Blight, unpubl data	Mandarte Island	Haro Strait, BC	2008, 2009	Egg size	2
20	LK Blight, unpubl data	Arbutus Island	Haro Strait, BC	2010	Lay date	1

1 See Acknowledgements for list of contributing museums.

2 See Table S1 for colony details.

3 See text.

4 WA – Washington, USA.

5 BC – British Columbia, Canada.

### Statistical analyses

I used meta-analysis rather than a standard statistical approach because disparate datasets derived from a group of primary studies must be properly weighted to yield correct standard errors and *p*-values and meta-analysis has been developed specifically to perform these weightings correctly, increasing the power of significance tests while retaining robustness [45], [46]. I used meta-regression, with fit assessed using *Q*-tests [45], [46], to analyse trends in glaucous-winged gull egg and clutch size over time and to examine the relationship between clutch size and first egg date. I used random-effects meta-analytical models as these assume that component studies differ not only by within-study sampling error (as fixed-effects models do), but also by a genuine difference in effect sizes among studies [45], [46]. Random-effects models thus incorporate among-study (here, equivalent to inter-year) variance in their estimates, and thereby generate wider confidence intervals and more conservative results than do fixed-effect models. All meta-analyses require that the results of each study be distilled to a measure of the magnitude of the effect of the measured variable – the “effect size”. As I wished to ask whether egg and clutch size had decreased over time in response to declining availability of food, the effect sizes selected here for meta-analysis were mean annual egg volume and number of eggs per clutch. Variance is required to compute meta-analytical weightings and was provided in publications or calculated from raw data for all but five annual means of egg sizes, and one study reporting clutch size; for these, I imputed standard deviation (SD) from the pooled SD from all raw data for the study [47], using the formula
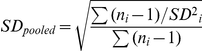
and egg volume was calculated as
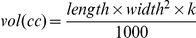
where length and width are in mm and k is the constant 0.476, determined by Harris [48] for another *Larus* gull.

I used the statistical software package Comprehensive Meta-Analysis v. 2.0 to perform all weightings and meta-analyses [49]. Welch's analysis of variance (robust to unequal sample size and variance) was used to compare mean first egg date in historical vs. current studies (1959–1986; 2008–2010).

## Results

### Egg size

**Figure 1:**
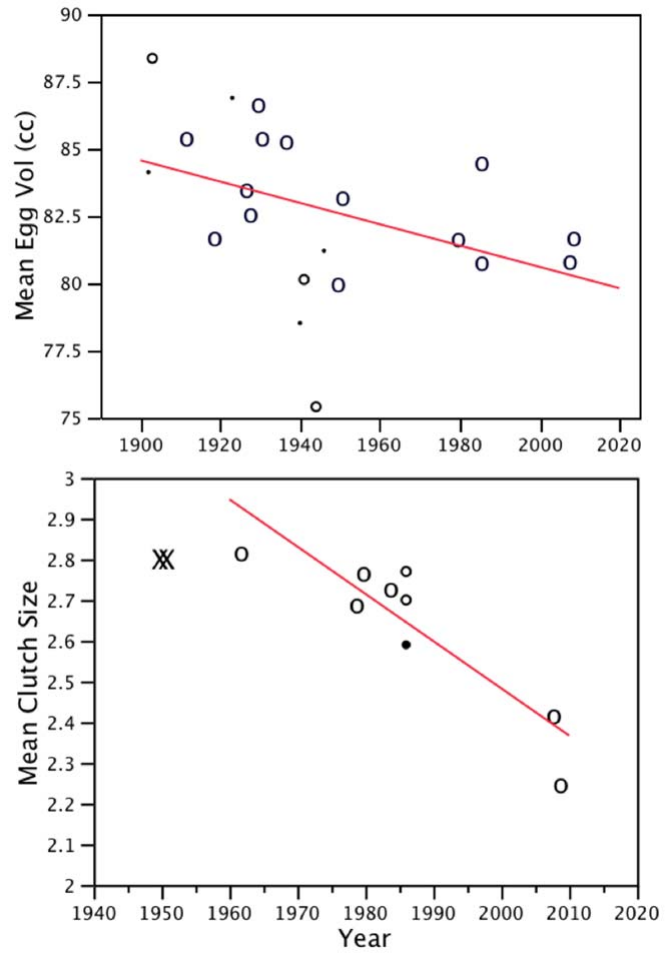
Meta-regression of glaucous-winged gull egg and clutch size vs. year, Salish Sea (SW Canada & NW USA). Meta-regression of glaucous-winged gull egg and clutch size vs. year, Salish Sea (SW Canada & NW USA). Symbol size represents meta-analytical weightings for each data point. Note different temporal scales on x-axes. (A) Egg volume decreased over the study period (1902–2009; *Q* = 7.211, *p*<0.01), with eggs now 5% smaller on average than at the turn of the 20^th^ century. (B) Clutch size decreased between 1962 and 2009 (*Q* = 27.30, *p*<0.001). Two data points from the 1940s–50s (represented by ×) are not included in the meta-analysis due to inadequate reporting criteria (see text), but are plotted here to further illustrate robustness of trend.

From 1902–2009, mean glaucous-winged gull egg volume decreased in the Salish Sea study area, with the random-effects model showing a significant negative relationship between year and egg volume (*Q* = 7.211; *p* = 0.007; Fig. 1a) and volume decreasing at an average of 0.04 cc y^−1^ (95% CI = −0.06–−0.01; egg volume range 78.52–88.36 cc; See Table S1 for a list of effect sizes) over the study period. This equates to an overall decrease of circa 5% (4.3 cc) in mean egg volume since 1902 (108 years).

### Clutch size and first egg date

**Figure 2:**
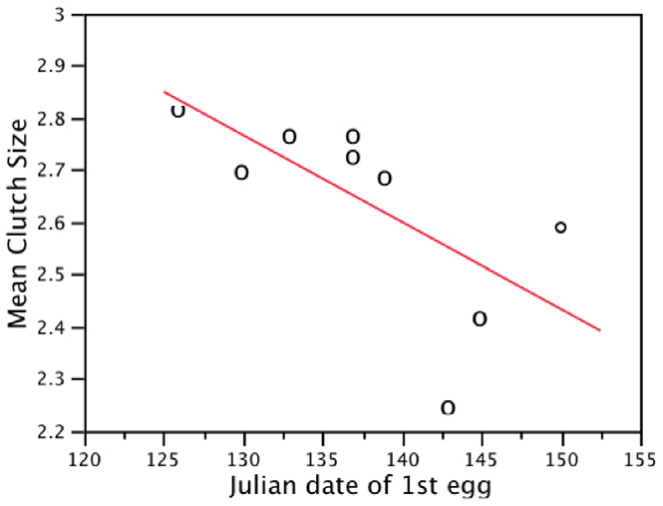
Meta-regression of glaucous-winged gull clutch size vs. year. Meta-regression of glaucous-winged gull clutch size vs. year. Clutch size decreased with delayed onset of breeding (first egg date; *Q* = 12.91, *p*<0.001; 1962–2009 data). Symbol size represents meta-analytical weightings for each data point.

**Figure 3:**
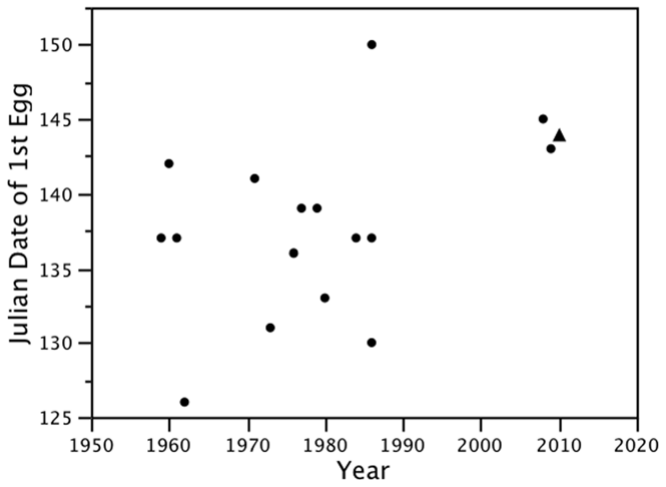
First egg date retreated significantly from 1959–2010. First egg date retreated significantly from 1959–2010 (*p* = 0.03, ▴: *n* = 2 observations), with mean commencement date 7 d later in 2008–2010 than in earlier decades (*F* = 20.12, *p*<0.001).

As with egg size, average clutch size decreased during the study period (*Q* = 27.30, *p*<0.001; Fig. 1B), declining from a mean of 2.82 eggs per clutch in 1962 to one of 2.25 in 2009 (Table S1). Though not included in the analysis, qualitative descriptions of clutch size from the 1950s are consistent with these results (Fig. 1B). There was a negative relationship between first egg date and mean clutch size in a given year (*Q* = 12.91, *p*<0.001; Fig. 2), with smaller clutches occurring in years where egg laying commenced later. Timing of clutch initiation also retreated over time. For historical data collected between 1959 and 1986 the mean first egg date was 15 May (range 4–28 May). From 2008–2010 the average first egg date was 22 May, 7 d later than in earlier decades (range 21–23 May; *F* = 20.12, *p*<0.001; Fig. 3).

## Discussion

### Egg size, clutch size and lay date

These results reveal long-term declines in egg and clutch sizes of glaucous-winged gulls in the Salish Sea, likely as a result of reductions in availability of food. Mean egg size decreased by circa 5% from 1902–2009. Similarly, mean clutch size has declined to the lowest ever recorded for the region. Five of nine studies reporting clutch size took place at a single site (Mandarte Island), including 21^st^ century clutch sizes, so that the Mandarte data may have had a large influence on the results. However, opportunistically-collected data from other Salish Sea colonies appear to support the hypothesis of a regional clutch size decline over time: population counts at 17 colonies recorded a mean clutch size of 2.29 in 2010 (LKB unpubl. data). Though these additional data represent only a snapshot of the number of eggs per nest (and were thus not incorporated into the analysis), they provide a good proxy for mean annual clutch size as they were collected immediately prior to hatching, when most gulls should be incubating an entire clutch. These concurrent egg and clutch size declines are noteworthy because although gulls lack an obligate clutch size, a mode of three is a well-known feature of most *Larus* gulls' biology, and egg size reduction is a flexible mechanism that allows birds to accommodate limited decreases in energy availability while maintaining offspring number [5]. Visual inspection of the egg and clutch size data suggests the possibility of an opposing strategy – the maintenance of somewhat larger eggs on average as clutch size began to decline – but post-1980 egg size data were too sparse to pursue this possibility. I suggest this study's egg and clutch size results are consistent with a decline in availability of high-quality fish prey pre- and during the breeding season. The actual cost of egg production to breeding birds in general is controversial [4] but for gulls at least there is good evidence that food input, particularly in the form of protein, affects egg size and clutch number [3], [6], [22], [29], [50], [51]. California gulls (*L. californicus*) breeding at Mono Lake, California have been reduced to laying 2-egg clutches since the early 1900s (with eggs also smaller than those from other populations); this is apparently due to regional food shortages [52]. In red-billed gulls (*L. novaehollandiae*), egg and clutch size over 41 years were positively correlated with the availability of their preferred prey, the euphausiid *Nyctiphanes australis* [53]. Decreasing egg and clutch sizes are predictable in growing populations of birds, a response hypothesised as being due to increased competition for food [54]–[56]. However, though this study population of glaucous-winged gulls grew through approximately the 1930s–1980s [57] it has subsequently been decreasing [58] but egg and clutch sizes have not increased in response.

Similarly, as predicted based on numerous other studies [6], I found a negative relationship between clutch size and first egg date, with smaller clutches produced on average in years when laying commenced later. The relationship between food supply and lay date in birds is well established, including in some gull populations [6], [7], [53], [59]. Gulls are capital breeders that, like many waterbirds, depend partly on endogenous reserves acquired prior to initiation of breeding [27], [60]. A primary source of late winter and early spring food for gulls as well as other waterbirds in the study region has been the considerable influx of nutrients provided by the sequential spawning of herring at sites along the north-eastern Pacific coast [61], [62]. For example, surf (*Melanitta perspicillata*) and white-winged scoter (*M. fusca*) mass gains in March and April are related to presence of spawning herring [63]. However, stock declines and temporal contraction of spawning herring in the Salish Sea (most herring there now spawn in March) [13], [24] means that access to this prey resource has declined for pre-breeding gulls over at least the past 40 years; other forage fishes such as pilchard (*Sardinops sagax*) were rendered commercially extinct in the study area as early as the 1940s [11]. A decrease in Salish Sea herring size-at-age suggests a possible decline in quality as well as availability of this favoured prey since the 1970s, and declines in forage fish food value has been shown to negatively affect seabird productivity in other systems [26].

First egg dates of glaucous-winged gulls have become later since 1959, from a mean date of 15 May in previously-published literature (1959–1986) to one of 22 May in my 2008–2010 field study. This response is largely unexpected in terms of global trends as breeding season phenology has been advancing in the majority of bird species studied worldwide, with a relationship found between lay date and climate [64]–[66]. While most seabirds examined in other studies also demonstrate advancing laying dates, their responses have been more variable, with some species or populations instead exhibiting significant delays in initiation of breeding over recent decades, and warming sea surface temperature (SST) invoked to explain both advancing and delaying trends [66], [67]. It is therefore possible that gulls' delayed lay dates are a response to changing climate. However, I found no relationship between glaucous-winged gull first egg date and local mean annual SST (from archived data recorded at Race Rocks Lighthouse Station, 48.30° W, 123.53° N; *F* = 0.006, *p* = 0.94) for the years over which phenological data were available, despite a warming trend in regional SSTs since 1970 [68]. The observed delay in laying thus supports the hypothesis of gulls responding to an overall food decline, rather than to climate. Delayed laying has also been associated with food availability in other larids, e.g., red-billed gulls laid later when euphausiid availability was low.

This study shows that glaucous-winged gull egg and clutch size have decreased over time in the Salish Sea, but these changes are biologically unimportant if lifetime reproductive success is unaffected. Though I lacked the data to analyse reproductive success per se over time, my results are suggestive of biologically meaningful changes that may in part explain ongoing population declines [58]. The most important effect of increased egg size in birds overall seems to be improved survival in the days post-hatching, allowing young chicks to weather temporary food shortages [5], [7], [69], [70]. However, evidence from multiple studies also shows egg size to be positively related to hatching success, growth rate and chick survival [71]. The relationship between egg size and ongoing fitness seems best established in seabirds [70] with a handful of studies demonstrating that egg size is correlated with overall reproductive success and that chick size at fledging affects future survival [53], [72], [73]. Based on the importance of high-quality fish prey during egg formation, and the egg size, clutch size and lay date patterns documented here, I hypothesise that recent marine food web changes may be affecting gull population dynamics in the Salish Sea study area. Though reduced access to trash via modern landfill management practices may conceivably have also affected some aspects of this population's dynamics over time, it appears likely that forage fish declines are playing an important role in recent population declines that are trending toward early 1900 levels, when gull numbers were locally depressed by egging and persecution [74]. This response hints at the potential for limits to the resilience of even generalist foragers.

### Alternative hypotheses

While food-related explanations are the most parsimonious for trends observed here, other possible causes exist. For example, pollutants such as PCBs and PBDEs also affect avian reproduction including egg and clutch size in birds [72]. It is unlikely that contaminants are a causative factor here, however, as DDE and other chlorinated hydrocarbons levels have mostly decreased in eggs of avian indicator species in the region since the late 1970s [75], [76]. Though other contaminants such as PBDEs are increasing, their occurrence is more recent (since the 1980s; [77]), and thus out of phase with observed egg and clutch declines. Two recent studies have documented body size declines and morphological changes in North American birds over the past 50–100 y, likely related to climate change [78], [79]; body size changes might also affect reproductive output. I was unable to rule out this explanation and suggest it would be a fruitful direction for further study, but note that female body size explains only a small proportion of egg size variability [7]. The Salish Sea also lies within the glaucous-winged gull × western gull hybrid zone [37], and it may be that increasing introgression of western gull genes has been altering the foraging ecology of the Salish Sea population, leading to ongoing effects on egg and clutch size. However, in the Canadian portion of the Salish Sea at least, western gull introgression does not yet appear well-advanced, with the region far beyond the edge of the hybrid zone [37] and only a handful of obvious hybrids (i.e., birds with dark-coloured primaries) observed in a 2010 survey of the breeding glaucous-winged gull population (LKB, pers. obs.). Finally, direct and indirect pressures from increasing numbers of bald eagles (*Haliaeetus leucocephalus*) have been suggested as a factor in gull population declines in the region [58], and it may be that a shift in energy allocation from egg production to increased vigilance has led to declining egg and clutch sizes. Though egg size declines are out of synch with eagle population increases [58], this nonetheless represents an interesting possibility that remains to be explored.

### Conclusions

Birds should ultimately alter reproductive traits and phenology to respond to shifts in underlying features of their food webs. There is experimental evidence for supplemental food increasing gull egg and clutch size in years of poor food availability, but not in good years, indicating the ultimate limits to reproductive output as well as the potential for proximate adjustments based on diet [80]. Nutritional requirements prior to egg laying (and possibly during certain phases of chick rearing; [21]) are likely precise and may require birds to consume high quality fish prey at this time. Thus, glaucous-winged gulls may be unable to use alternative food sources (e.g., trash) to buffer against consistent shortages of natural foods during certain periods of their breeding cycle, and could be undergoing an ongoing trade-off of their own survival against production of offspring. It is possible that the study population may be shifting toward a modal 2-egg clutch, as has occurred in another food-limited population of gulls in the 20^th^ century [52]. Experimentally testing whether gulls in the Salish Sea respond to increased high-quality fish prey by increasing egg or clutch size would provide more conclusive evidence for or against natural food supply as a mechanism driving observed trends. Though glaucous-winged gulls are generalist feeders that are expected to buffer themselves against ecological change, the shifts in reproductive traits identified here suggest a significant impoverishment of a coastal marine ecosystem bordering one of the most rapidly growing areas in North America. Interestingly, in 2008 glaucous-winged gull egg and clutch size (and reproductive success; LKB unpubl. data) remained low despite north-eastern Pacific waters being the coolest in 50 years of records and productivity being the highest ever viewed via satellite in August [25], suggesting that the study area's coastal sea may be more strongly affected by regional than by basin-wide factors (cf. [12]). Future studies should investigate details of long-term trends in gull diet, possibly using a stable isotope approach. Finally, I suggest that eggs in museum collections represent an underutilised resource for observing effects of environmental change on avian demography over time.

### Ethics Statement

Field research contributing to this study was carried out under Permit No. A07-0309 from the University of British Columbia's Animal Care Committee, and under Research Permit No. BC-10-0057 from the Canadian Wildlife Service. Collection of author-generated data was carried out using protocols that explicitly minimised disturbance to breeding birds.

## Supporting Information

Table S1 Summary of all effect sizes used in meta-analyses of changes in egg and clutch size over time.
(DOC)
