# Supplementary material for: Egg Production in a Coastal Seabird, the Glaucous-Winged Gull (Larus glaucescens), Declines during the Last Century
Source: PLoS One. 2011 Jul 18;6(7):e22027. doi: 10.1371/journal.pone.0022027 (PMC3138773; doi:10.1371/journal.pone.0022027)
Supplement: Table S1 — Summary of all effect sizes used in meta-analyses of changes in egg and clutch size over time. (DOC) [file pone.0022027.s001.doc]

Table S1. Summary of all effect sizes used in meta-analyses of changes in egg and clutch size over time.

| Data ID | Data source | Study location | Study year | Variable | Effect size (mean egg or clutch size) | SD | *N* (samples per study or year) |
| --- | --- | --- | --- | --- | --- | --- | --- |
| Mus1902 | Museum collections | Mandarte I, BC* | 1902 | Egg size | 84.13 | 2.07 | 6 |
| Mus1903 | Museum collections | Mandarte I, BC | 1903 | Egg size | 88.36 | 7.20 | 13 |
| Mus1912 | Museum collections | Island County, WA;* Whidbey I, WA; Williamson Rock, WA | 1912 | Egg size | 85.44 | 6.41 | 39 |
| Mus1919 | Museum collections | Bird Rock, WA; Flattop I, WA; White Rock, WA | 1919 | Egg size | 81.73 | 6.23 | 24 |
| Mus1923 | Museum collections | Orcas I, WA; Peapod I, WA | 1923 | Egg size | 86.89 | 6.6 | 6 |
| Mus1927 | Museum collections | Gull Rock, WA; Viti Rocks, WA | 1927 | Egg size | 83.54 | 7.27 | 50 |
| Mus1928 | Museum collections | San Juan Islands, USA; | 1928 | Egg size | 82.62 | 8.28 | 23 |
| Mus1930 | Museum collections | Viti Rocks, WA; Williamson Rock, WA | 1930 | Egg size | 86.67 | 5.48 | 38 |
| Mus1931 | Museum collections | Viti Rocks, WA | 1931 | Egg size | 85.42 | 6.32 | 42 |
| Mus1937 | Museum collections | Colville Rock, WA; Williamson Rock, WA | 1937 | Egg size | 85.32 | 7.96 | 59 |
| Mus1940 | Museum collections | Gulf of Georgia, BC; Howe Sound, BC | 1940 | Egg size | 78.52 | 6.88 | 6 |
| Mus1941 | Museum collections | Howe Sound, BC | 1941 | Egg size | 80.14 | 6.64 | 12 |
| Mus1944 | Museum collections | Howe Sound, BC | 1944 | Egg size | 75.41 | 4.17 | 6 |
| Mus1946 | Museum collections | Howe Sound, BC | 1946 | Egg size | 81.21 | 6.36 | 6 |
| Schultz | Schultz 1951 | San Juan Is, WA | 1948 | Egg size | 80.03 | 7.64 | 43 |
| J-V&Booth | James-Veitch & Booth 1954 | Williamson Rock, WA | 1951 | Egg size | 83.23 | 7.64 | 30 |
| Verbeek | Verbeek 1986 | Mandarte I, BC | 1980 | Egg size | 81.69 | 7.64 | 144 |
| VermeerCol | Vermeer 1988 | Vancouver Harbour, BC | 1986 | Egg size | 80.81 | 7.64 | 62 |
| VermeerSol | Vermeer 1988 | Vancouver Harbour, BC | 1986 | Egg size | 84.50 | 7.64 | 26 |
| Mandarte08 | LKB unpubl data | Mandarte I, BC | 2008 | Egg size | 80.84 | 8.02 | 345 |
| Mandarte09 | LKB unpubl data | Mandarte I, BC | 2009 | Egg size | 81.73 | 7.98 | 267 |
| VermeerCS | Vermeer 1963 | Mandarte I, BC | 1962 | Clutch size | 2.82 | 0.40 | 479 |
| Verbeek1986a | Verbeek 1986 | Mandarte I, BC | 1979 | Clutch size | 2.69 | 0.59 | 297 |
| Verbeek1986b | Verbeek 1986 | Mandarte I, BC | 1980 | Clutch size | 2.77 | 0.47 | 417 |
| Reid | Reid 1987 | Protection I, WA | 1984 | Clutch size | 2.73 | 0.78 | 704 |
| Vermeer1988a | Vermeer 1988 | Vancouver Harbour, BC | 1986 | Clutch size | 2.70 | 0.60 | 80 |
| Vermeer1988b | Vermeer 1988 | Vancouver Harbour, BC | 1986 | Clutch size | 2.77 | 0.51 | 52 |
| Hooper | Hooper 1988 | Victoria Harbour, BC | 1986 | Clutch size | 2.59 | 0.79 | 22 |
| Mandarte2008 | LKB unpubl data | Mandarte I, BC | 2008 | Clutch size | 2.42 | 0.73 | 210 |
| Mandarte2009 | LKB unpubl data | Mandarte I, BC | 2009 | Clutch size | 2.25 | 0.84 | 175 |

* BC – British Columbia, Canada ; WA – Washington, USA;
